# Supplementary material for: An exploratory survey about using ChatGPT in education, healthcare, and research
Source: medRxiv. 2023 Apr 3:2023.03.31.23287979. Preprint. [Version 1] doi: 10.1101/2023.03.31.23287979 (PMC10104227; doi:10.1101/2023.03.31.23287979)
Supplement: Supplement 1 [file media-1.pdf]

Supplemental Document

Table 1. Respondents' selections, grouped by respondent role.

			Grouped by Role					
		Overall	Medical Student, Resident, Fellow	Graduate Student, Postdoc Researcher	Clinical Faculty	Research Faculty	Administrative Staff	Other
**n**		420	14	53	45	65	70	173
**Used_before, n (%)**	No	252 (60.0)	5 (35.7)	23 (43.4)	31 (68.9)	33 (50.8)	48 (68.6)	112 (64.7)
	Yes	168 (40.0)	9 (64.3)	30 (56.6)	14 (31.1)	32 (49.2)	22 (31.4)	61 (35.3)
**Interested, n (%)**	Not at all	19 (4.5)		2 (3.8)		5 (7.7)	4 (5.7)	8 (4.6)
	Very little	85 (20.2)	2 (14.3)	6 (11.3)	7 (15.6)	12 (18.5)	16 (22.9)	42 (24.3)
	Somewhat	209 (49.8)	3 (21.4)	27 (50.9)	22 (48.9)	29 (44.6)	39 (55.7)	89 (51.4)
	To a great extent	107 (25.5)	9 (64.3)	18 (34.0)	16 (35.6)	19 (29.2)	11 (15.7)	34 (19.7)
**Education, n (%)**	No, it should be banned	11 (2.6)	1 (7.1)		1 (2.2)	1 (1.5)	2 (2.9)	6 (3.5)
	I don't know, it is too early to make a statement	226 (53.8)	4 (28.6)	23 (43.4)	24 (53.3)	36 (55.4)	39 (55.7)	100 (57.8)
	Yes, it should be actively incorporated	183 (43.6)	9 (64.3)	30 (56.6)	20 (44.4)	28 (43.1)	29 (41.4)	67 (38.7)

**Research, n (%)**	No, it should not be used at all	6 (1.4)				3 (4.6)	1 (1.4)	2 (1.2)
	I don't know, it is too early to make a statement	75 (17.9)	1 (7.1)	4 (7.5)	12 (27.3)	6 (9.2)	14 (20.0)	38 (22.1)
	Yes, but it should only be used to help brainstorm	68 (16.3)		17 (32.1)	5 (11.4)	8 (12.3)	10 (14.3)	28 (16.3)
	Yes, as long as its use is transparently disclosed	259 (62.0)	12 (85.7)	28 (52.8)	26 (59.1)	46 (70.8)	44 (62.9)	103 (59.9)
	Yes, disclosure is NOT needed	10 (2.4)	1 (7.1)	4 (7.5)	1 (2.3)	2 (3.1)	1 (1.4)	1 (0.6)
**Healthcare, n (%)**	No, it should not be used at all	15 (3.6)		1 (1.9)	1 (2.3)	1 (1.5)	5 (7.1)	7 (4.0)
	I don't know, it is too early to make a statement	177 (42.2)	1 (7.1)	15 (28.3)	22 (50.0)	25 (38.5)	27 (38.6)	87 (50.3)
	Yes, it can be used for administrative purposes	176 (42.0)	10 (71.4)	25 (47.2)	19 (43.2)	27 (41.5)	33 (47.1)	62 (35.8)
	Yes, it can be used for any purpose	51 (12.2)	3 (21.4)	12 (22.6)	2 (4.5)	12 (18.5)	5 (7.1)	17 (9.8)

Survey delivered via Slido.

1. **What's your current role?**
    - Medical Student, Resident, Fellow
    - Graduate Student, Postdoc Researcher
    - Clinical Faculty
    - Research Faculty
    - Administrative Staff
    - Other
2. **Have you used ChatGPT?**
    - Yes
    - No
3. **How interested are you in using ChatGPT in your day to day work?**
    - To a Great Extent
    - Somewhat
    - Very Little
    - Not at All
4. **Can ChatGPT be used in education?**
    - No, it should be banned
    - Yes, it should be actively incorporated
    - I don't know, it is too early to make a statement
5. **Can ChatGPT be used for science?**
    - No, it should not be used at all
    - Yes, but it should only be used to help brainstorm
    - Yes, as long as its use is transparently disclosed
    - Yes, disclosure is NOT needed
    - I don't know, it is too early to make a statement
6. **Can ChatGPT be used in healthcare?**
    - No, it should not be used at all
    - Yes, it can only be used to help write administrative content such as emails to insurance companies or to patients
    - Yes, it can be used for any purpose
    - I don't know, it is too early to make a statement
7. **Using one keyword, describe challenges of using ChatGPT**
8. **Using one keyword, describe benefits of using ChatGPT**
